# Electrically Interconnected Platinum Nanonetworks for Flexible Electronics

**DOI:** 10.1021/acsomega.5c00237

**Published:** 2025-03-11

**Authors:** Sherjeel Mahmood Baig, Hideki Abe

**Affiliations:** † National Institute for Materials Science, 1-1 Namiki, Tsukuba, Ibaraki 305-0044 Japan; ‡ Graduate School of Science and Technology, Saitama University, 255 Shimookubo, Saitama 338-8570, Japan

## Abstract

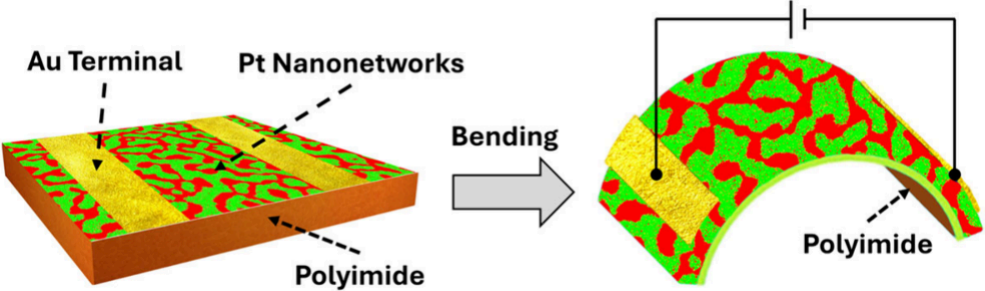

Flexible electronics are attracting attention due to the growing demand for lightweight, bendable devices that can conform to various surfaces including human skin. Although indium tin oxide (ITO) is widely used for electrical interconnection in flexible electronics, its brittleness limits its durability under repeated bending. Here, we introduce platinum (Pt) nanonetworks as an alternative to ITO, offering superior electrical stability under intense and repeated bending conditions. Electrically interconnected Pt nanonetworks with an average thickness below 50 nm are fabricated on polyimide (PI) substrates via an atmospheric treatment that promotes nanophase separation in thin deposition films of a platinum–cerium (Pt–Ce) alloy, developing a nanotexture of Pt and insulating cerium dioxide (CeO_2_). The resulting Pt nanonetworks on PI exhibit high mechanical flexibility, maintaining a sheet resistance of approximately 2.76 kΩ/sq even after 1000 bending cycles at varying diameters, down to 1.5 mm. Detailed characterization reveals critical temperature and time thresholds in the atmospheric treatment necessary to form interconnected Pt nanonetworks on solid surfaces: interconnected nanonetworks develop at lower temperatures and shorter treatment times, while higher temperatures and longer treatments lead to disconnected Pt nanoislands. LCR (Inductance, Capacitance, and Resistance) measurements further show that the interconnected Pt nanonetworks exhibit inductor-like electrical responses, while disconnected Pt nanoislands display capacitor-like behavior.

## Introduction

Flexible electronics have become a critical field due to the growing demand for lightweight, bendable, and stretchable devices that can be seamlessly integrated with various surfaces including human skin. From wearable health monitoring systems to foldable displays and touchscreens, flexible electronics require electroconductive materials capable of maintaining reliable performance even under significant mechanical strain. Traditional rigid materials, such as silicon (Si), are unsuitable for these applications due to their limited flexibility and vulnerability to mechanical failure.^1^ Indium tin oxide (ITO), commonly used for electric interconnections in flexible displays, is brittle,^2^ prone to cracking,^3^ And faces challenges due to the scarcity of indium, which hinders large-scale development.^4,5^ Dong et al. utilized laser interference lithography (LIL) to create ITO nanopatterns that enable multiaxial bending while maintaining low electric resistance.^6^ However, LIL is a complex, time-consuming, and low-throughput method.

Metal nanomesh or nanonetworks have garnered increasing attention for their ability to combine mechanical flexibility with high electrical conductivity. Seo et al. demonstrated that gold (Au) nanomesh fabricated via nanosphere lithography outperformed ITO interconnections in electrophysiology applications, offering higher flexibility.^7^ Guo et al. fabricated Au nanomesh on a flexible polydimethylsiloxane (PDMS) substrate using grain boundary lithography, a bilayer lift-off metallization process.^8^ However, their methods remain complex and time-consuming, requiring multiple lithographic steps. Adrien and colleagues fabricated Au nanomesh on polyethylene terephthalate (PET) substrates via a chemical process, bypassing lithography, and showed good electrical conductivity and exceptional stability under mechanical deformation.^9^ Unfortunately, their method has significant drawbacks, such as limitations in scalability due to the reliance on floating microdomains for nanomesh transfer and safety concerns related to the handling of concentrated nitric acid vapors during the dealloying process.

**Figure 1:**
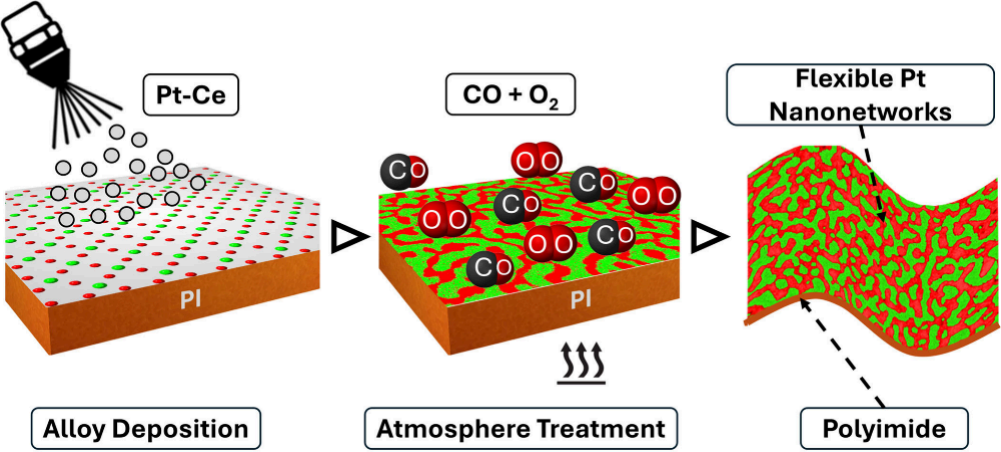
Schematic image for a device incorporating flexible platinum nanonetworks. Pt–Ce alloy was deposited over the polyimide surface. Followed by the atmosphere treatment in the presence of CO and O_2_ at the elevated temperature. The Pt nanonetworks (red) emerged via the oxidation of Ce into CeO_2_ (green) over the PI substrate.

Here, we present a straightforward method for fabricating flexible platinum (Pt) nanonetworks not only on solid substrates but on flexible substrates as well (Figure 1). The process begins with the deposition of 50 nm-thick platinum–cerium (Pt–Ce) alloy films onto the substrate surface, followed by an atmospheric treatment at elevated temperatures using a gas mixture of carbon monoxide (CO) and oxygen (O_2_). This treatment drives phase separation in the Pt–Ce alloy, forming a nanotexture of Pt and cerium dioxide (CeO_2_). This method is applicable to a range of precious metals, exhibiting similar nano structuring behavior with rare earth elements.^10−12^ The optimal conditions for producing highly interconnected Pt nanotextures were determined through a phase diagram created from atmospheric treatments applied to Pt–Ce films on silicon substrates, varying in temperature from 300–500 °C and treatment duration. Based on the phase diagram, electrically interconnected Pt nanonetworks were successfully fabricated on polyimide (PI) film substrates. These Pt nanonetworks on PI demonstrated outstanding electrical stability, maintaining a sheet resistance of 2.76 kΩ/sq even after 1000 bending cycles at a radius as small as 1.5 mm. The proposed fabrication method for Pt nanonetworks offers a practical approach to flexible electronics by using a simple protocol involving alloy film deposition and atmospheric treatments, enabling large-area electric interconnections without the need for complex processes or specialized equipment such as lithographs.

## Results and Discussion

**Figure 2:**
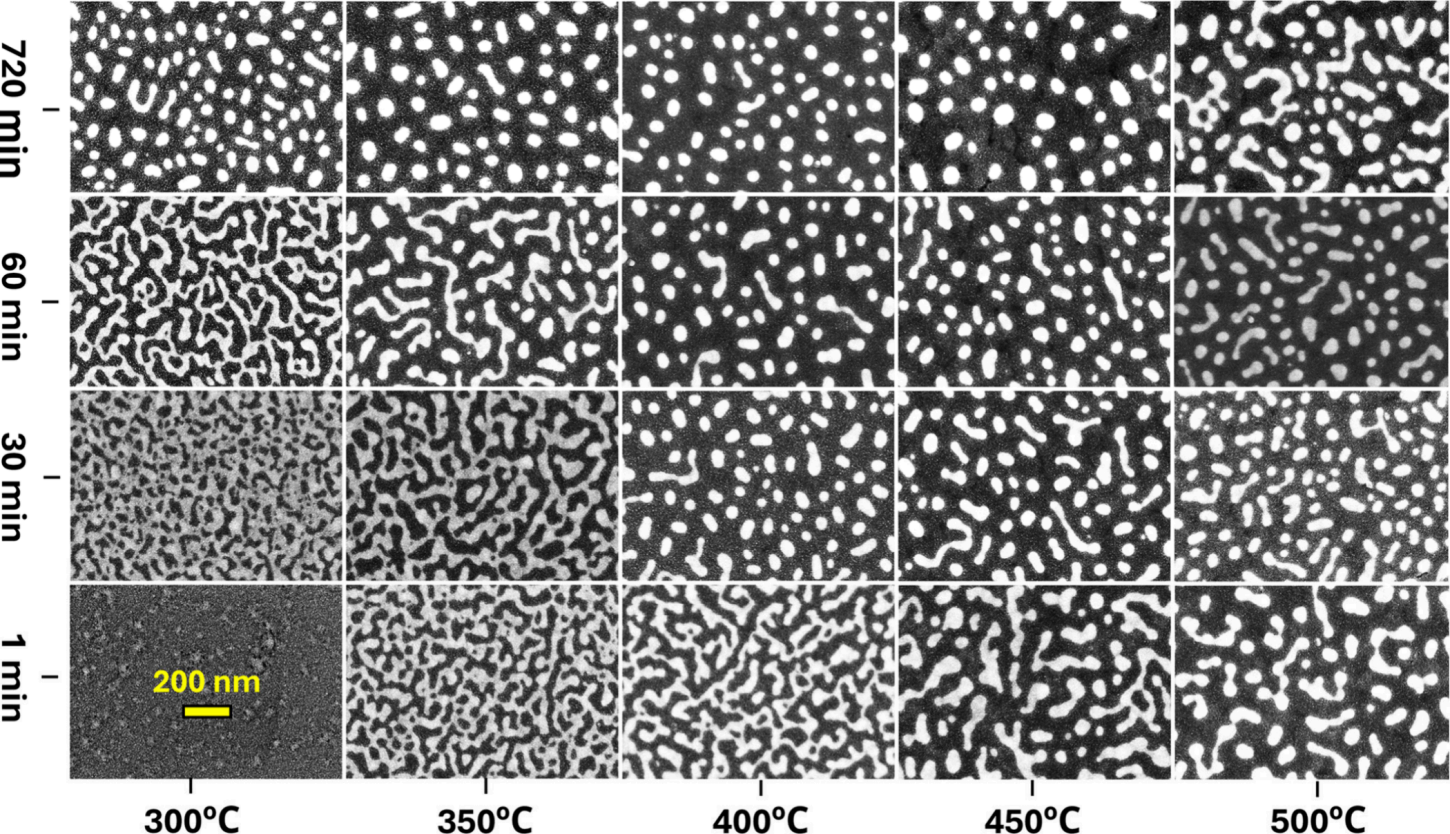
Phase diagram for atmospheric-treated Pt–Ce films, showing the morphological transition from interconnected networks to discrete nanoislands, as influenced by varying treatment temperatures (horizontal axis) and durations (vertical axis). The bright and dark areas correspond to the Pt and CeO_2_ phases, respectively.

Figure 2 presents a phase diagram illustrating the nanotextures of Pt–Ce films on Si substrates, subjected to varied atmospheric treatments. FE-SEM images of these films are arranged by treatment temperature and duration, showing the impact of these conditions on nanotexture morphology. The films were exposed to a CO, O_2_, and Ar gas mixture in a mole ratio of 2:1:97, with both temperature and treatment duration varying. An interconnected Pt nanotexture developed within the triangular region defined by the line from a treatment temperature of 400 °C and a duration of 60 min to the origin. The highest degree of interconnection appeared at 300 °C with a 30 min duration, whereas no pattern was observed at 300 °C with only a 1 min treatment. This suggests that temperatures below 300 °C or shorter durations lack the thermal energy needed to initiate the phase separation of Pt–Ce alloys required for forming the Pt and CeO_2_ nanotexture.

The Pt and CeO_2_ nanotexture underwent a morphological transition from interconnected Pt networks to isolated, discrete Pt islands as the treatment temperature exceeded 450 °C. This transition from networks to islands is attributed to the accelerated oxidation of Ce in the Pt–Ce alloy to CeO_2_ at elevated temperatures, which further promotes Pt atom agglomeration, disrupting the connectivity of the Pt nanonetworks. Extended treatment durations also led to the formation of Pt islands, indicating increased Pt agglomeration over time. In contrast, shorter treatment durations limited Pt atom diffusion and clustering at all temperatures, thereby preserving the enhanced connectivity within the Pt nanonetwork.

**Figure 3:**
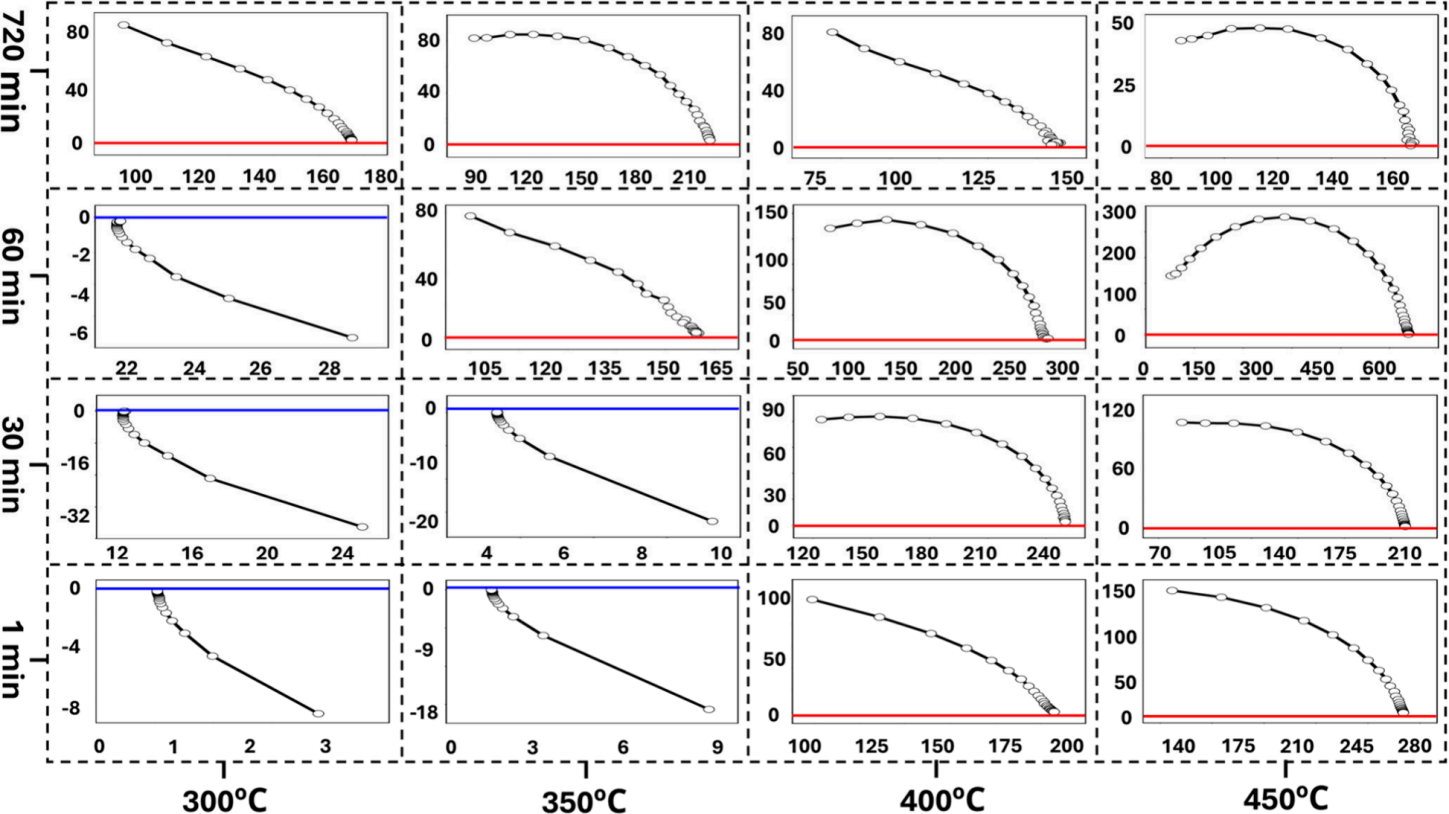
Nyquist plots for atmospheric-treated Pt–Ce films (see Figure 2), obtained from LCR measurements across a frequency range of 1–5 MHz. Cole–Cole arcs for interconnected nanotextures (blue baselines) are located in the lower half of the complex impedance plane, while arcs for island-like nanotextures (red baselines) appear in the upper half. Each Nyquist plot shows the impedance data, with the real and imaginary components plotted on the *x*-axis and *y*-axis, respectively, both in ohms (Ω).

LCR measurements offered valuable insights into the relationship between electrical properties and the morphology of the Pt nanotextures. LCR measurements were performed over a frequency range of 1 Hz to 5 MHz. Each of the Nyquist plots in Figure 3 corresponds to the FE-SEM images in Figure 2. The interconnected Pt nanonetworks, observed at lower temperatures for short durations in Figure 2, exhibited inductor-like electrical behavior, as shown in Figure 3. The Cole–Cole arcs for these networks were positioned on the lower side of the complex impedance plane, typical for a parallel configuration of an inductor and resistor. By fitting the Cole–Cole arc with an equivalent circuit (Figure S1), the inductance and sheet resistance of the Pt nanonetworks were calculated to be 0.7 μH and 2.76 kΩ/sq, respectively. The increase in sheet resistance was observed as compared to the as-deposited Pt–Ce film (0.6 kΩ/sq) due to the less uniform structure, localized CeO_2_ regions, and nanoscale current confinement.

The Pt nanoislands observed at temperatures between 300 and 450 °C for extended durations exhibited capacitor-like responses, in contrast to the inductor-like behavior of Pt nanonetworks. The Cole–Cole arcs for the Pt islands appeared on the upper half of the complex impedance plane, characteristic of a parallel circuit comprising a capacitor and resistor (Figure S2). The capacitance of the Pt islands was calculated to range from 0.2 to 0.6 nF, while the sheet resistance was 30 kΩ/sq. These Pt nanoislands, separated by insulating CeO_2_, store charge between neighboring islands, but are unsuitable for applications involving direct current due to their high sheet resistance. The frequency-dependent response of each sample, along with the corresponding Z-fitted curves, is displayed in Figure S3. Impedance analysis up to 30 MHz confirmed that inductive nanonetworks and capacitive nanoislands retained stability, even under extreme frequency testing (Figure S4 and S5).

**Figure 4:**
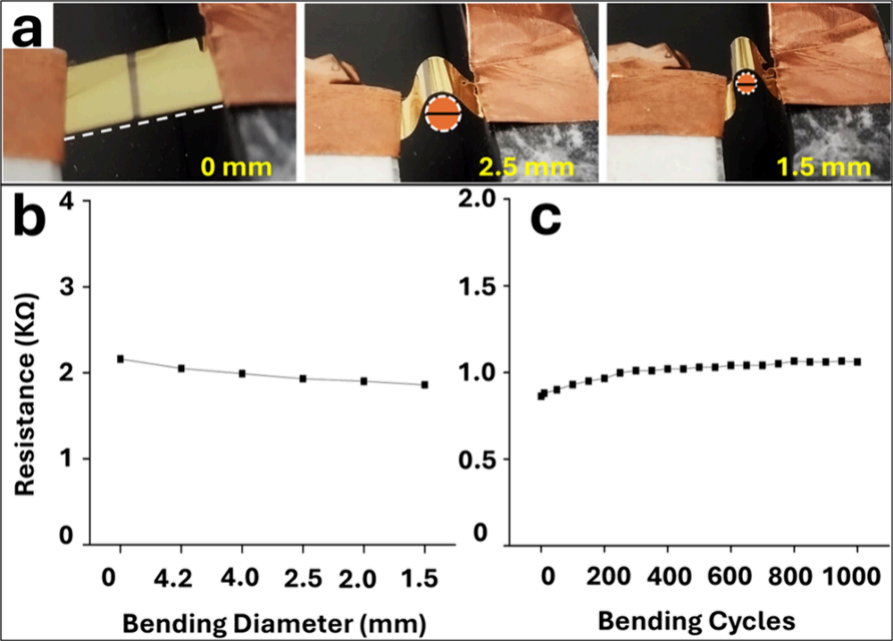
(a) Bending setup and connection terminals, with conductive tape contacting Au–Ti terminals. (b) Flexibility test showing the sheet resistance of the Pt nanonetwork sample at bending diameters down to 1.5 mm. (c) Cyclic bending test results for the Pt nanonetwork sample, demonstrating sheet resistance stability over 1000 cycles of bending at a diameter of 1.5 mm. Two different samples were utilized for panels (b) and (c).

Flexibility tests were conducted on Pt nanonetworks on polyimide films by subjecting them to repeated bending at various diameters (4.2 mm, 4.0 mm, 2.5 mm, 2.0 mm, and 1.5 mm), as shown in Figure S6. The bending setup and connection terminals are illustrated in Figure 4a, where conductive tape was used over the Au–Ti terminals to ensure smooth and nondestructive connections. Sheet resistance was measured at different bending diameters, revealing that the sheet resistance of the Pt nanonetworks on polyimide films remained approximately constant even after 1000 bending cycles at a bending radius as small as 1.5 mm. While the as-deposited 50 nm Pt–Ce alloy on PI showed a nearly doubling of sheet resistance after only 20 bending cycles at a 1.5 mm radius, indicating significant brittleness (see Figure S7). Although minor resistance increase observed in Pt nanonetworks due to Au–Ti terminal cracking (see FE-SEM images in Figure S8 and S9). On the other hand, the 50 μm thick PI film (substrate), with a 1.5 mm bending radius, experiences 0.017% strain, which does not impact the excellent flexibility of Pt nanostructures. Our Pt nanonetwork’s behavior is significantly better than that of traditional ITO thin films, which typically experience a rapid increase in resistance upon bending due to crack formation.

## Conclusions

We successfully demonstrated the fabrication of highly flexible and electrically interconnected Pt nanonetworks on solid substrates by a simple method using Pt–Ce alloy sputtering followed by atmospheric treatments. The Pt nanonetworks showed high electrical conductivity, mechanical flexibility, and excellent stability to repeated bending, serving as an ideal candidate material for flexible electronics. Furthermore, the proposed fabrication method is cost-effective, easy to implement, and does not require complex equipment, providing a promising approach for the scalable manufacturing of flexible electronic devices.

## Methods

### Alloy Synthesis and Characterization

A Pt–Ce alloy target was prepared by melting high-purity Pt (Furuya Kinzoku, 99.9%) and Ce (Aldrich, 99.9%) metal ingots in a mole ratio of Pt:Ce = 2:1 using an arc torch in a pure Ar (99.9999%) atmosphere. The phase purity of the resulting Pt_2_Ce alloy was confirmed via powder X-ray diffraction (*p*XRD, X’Pert Pro, Panalytical).^13^

### Alloy Thin Film Deposition on Si and Post-treatment

Thin films of the Pt–Ce alloy, 50 ± 1 nm thick, were deposited onto Si substrates (380 μm thick Si wafer) at room temperature (see FE-SEM and EDX images in Figure S10) using an electron-beam evaporator (MB-501010) (Figure S11). Atmospheric treatments were applied to the Pt–Ce films at temperatures ranging from 300 to 500 °C for durations of 1 min to 12 h, in a controlled O_2_ and CO atmosphere balanced with Ar at a volumetric ratio of 1:2:97 and a flow rate of 10 mL/min. The Pt–Ce films were converted into composite films of Pt and CeO_2_ through atmospheric treatments, forming different nanotextures depending on the treatment conditions.

### Thin Film Characterization

The nanotexture of the atmospheric-treated Pt–Ce films was analyzed to identify the optimal conditions for highly interconnected Pt nanonetworks. The analysis involved Grazing Incidence X-ray Diffraction (GIXRD) (Figure S12), Field Emission Scanning Electron Microscopy (FE-SEM), and Energy-Dispersive X-ray (EDX) analysis (Figure S13, and Atomic Force Microscopy (AFM) (Figure S14), supplemented by previously published data.^13^

### Nano Device Fabrication

Gold–titanium (Au–Ti) alloy terminals were deposited onto the atmospheric-treated films over Si substrate, leaving a 25 μm gap between terminals for inductance, capacitance, and resistance (LCR) measurements by the 2-probe method (Figures S15, S16).

### Alloy Thin Film Deposition on PI

Finally, Pt–Ce alloy thin films were deposited onto polyimide (PI) substrates using the same electron beam evaporator process, followed by atmospheric treatments under the optimal condition at 300 °C for 30 min (see FE-SEM images in Figures S17–S19). Au–Ti terminals were then deposited to the film (Pt-CeO_2_/PI) with a 1 mm gap between terminals using the electron-beam evaporator (MB-501010). The electrical resistance of the terminated film was quantified by the 4-probe method (Figure S20), subjected to repeated bending up to 1000 cycles at diameters as small as 1.5 mm.

## Supporting Information Available

Additional experimental details, characterization data, and supplementary images that support the findings in the main manuscript (PDF)
