# Supplementary material for: Electrically Interconnected Platinum Nanonetworks for Flexible Electronics
Source: ACS Omega. 2025 Mar 11;10(11):11562–6. doi: 10.1021/acsomega.5c00237 (PMC11947793; doi:10.1021/acsomega.5c00237)
Supplement: Supplementary file 1 — ao5c00237_si_001.pdf [file ao5c00237_si_001.pdf]

Supporting Information

Title:

Electrically Interconnected Platinum Nanonetworks for Flexible Electronics

Sherjeel Mahmood Baig*[1,2], Hideki Abe*[1,2]

1. National Institute for Materials Science, 1-1 Namiki, Tsukuba, Ibaraki, 305-0044 Japan.
2. Graduate School of Science and Technology, Saitama University, 255 Shimookubo, Saitama 338-8570, Japan.

List of Figures

Figure 1. Suggested equivalent circuit for the electronically interconnected Pt nanonetworks for the frequency range (0Hz to 3-5MHz). 4
Figure 2. Suggested equivalent circuit for the disconnected Pt nano islands for the frequency range (0Hz to 3-5MHz). 4
Figure 3. The frequency-dependent response of each sample (blue), along with their corresponding Z-fitted curves (red) for the frequency range (0Hz to 3-5MHz). 5
Figure 4. Suggested equivalent circuit for the electronically interconnected Pt nanonetworks for the frequency range (0Hz to 30MHz) 6
Figure 5. The frequency-dependent response of each sample (blue), along with their corresponding Z-fitted curves (red) for the frequency range (0Hz to 30MHz). 7
Figure 6. Real time images of flexible nanonetworks over polyimide substrate at different bending diameters. 8
Figure 7. FE-SEM images of PtCe alloy film over PI, (a) 0 bending cycle at scale 500 nm, after 20 bending cycles (b), at scales of 500nm and 5 um. 9
Figure 8. FE-SEM images of Au/Ti terminals at 0 bending cycles........................10
Figure 9. FE-SEM images of Au/Ti terminals after 1000 bending cycles. 10
Figure 10. FE-SEM and EDX images of as-deposited 50nm thin film of Pt-Ce alloy over Si substrate. 11
Figure 11. Electron beam evaporation mechanism 12
Figure 12. GIXRD of the as-deposited Pt-Ce film (left) and atmosphere-treated Pt-Ce film (right). 13
Figure 13. FE-SEM and EDX images of atmosphere-treated 50nm thin film of Pt-Ce alloy over the Si substrate. 14
Figure 14. AFM images of the atmosphere-treated 50nm thin films of Pt-Ce alloy over the Si substrate. 15
Figure 15. FE-Sem images of the gold-titanium (Au-Ti) terminals with a separation of 25 microns. 16
Figure 16. 2-Probe method setup for the impedance analysis 17
Figure 17. FE-SEM of as-deposited 50nm thin film of Pt-Ce alloy over the flexible PI substrate. 18
Figure 18. FE-SEM of atmosphere-treated 50nm thin film of Pt-Ce alloy over the flexible PI substrate. 18
Figure 19. EDX images of the atmosphere-treated 50nm thin film of Pt-Ce alloy over the flexible PI substrate. 19
Figure 20. 4-Probe method setup for the impedance analysis 20

Figure S1

The following circuit was suggested for our interconnected Pt nanonetworks in which the inherent inductive nature of the Pt nanonetworks, owing to the presence of the nanoloops is shwon parallel to the inherent resistance of the Pt nanonetworks which accounts for the the resistance offered by the interconnected Pt nanonetowrks to the flow of current. While the contact resistance has been shown in the series.

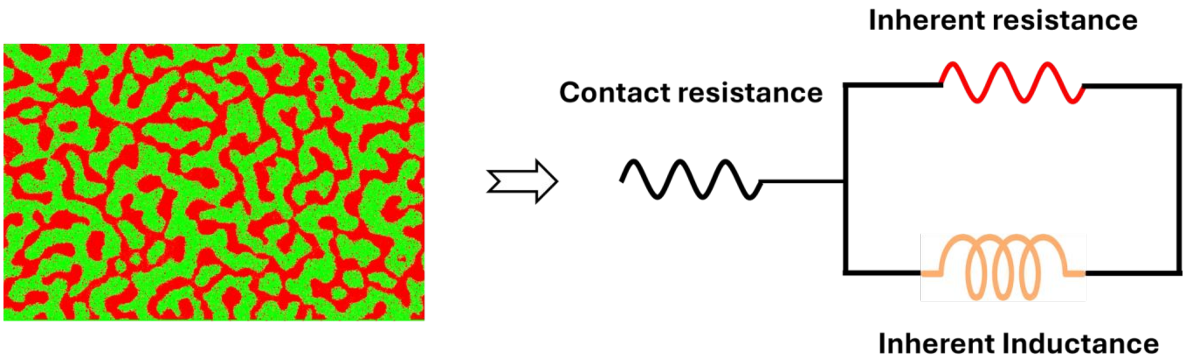

Figure 1. Suggested equivalent circuit for the electronically interconnected Pt nanonetworks for the frequency range (0Hz to 3-5MHz).

Figure S2

The following circuit was suggested for our disconnected Pt nano islands in which the inherent capacitive nature of the Pt nano islands is shwon parallel to the inherent resistance of the Pt nanonetworks. While the contact resistance has been shown in the series.

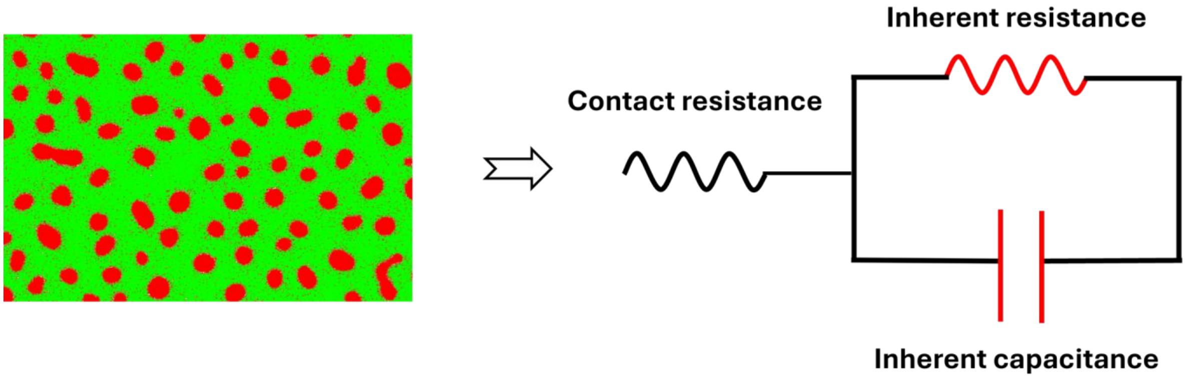

Figure 2. Suggested equivalent circuit for the disconnected Pt nano islands for the frequency range (0Hz to 3-5MHz).

Figure S3

The blue graph displays the original data collected from the samples at frequencies ranging from 0 Hz to 3-5 MHz. In contrast, the red graph represents the Z-fitting based on the equivalent circuit model as shown in Figure S11 and Figure S12. Each Nyquist plot shows the impedance data, with the real and imaginary components plotted on the x-axis and y-axis, respectively, both in ohms (Ω).

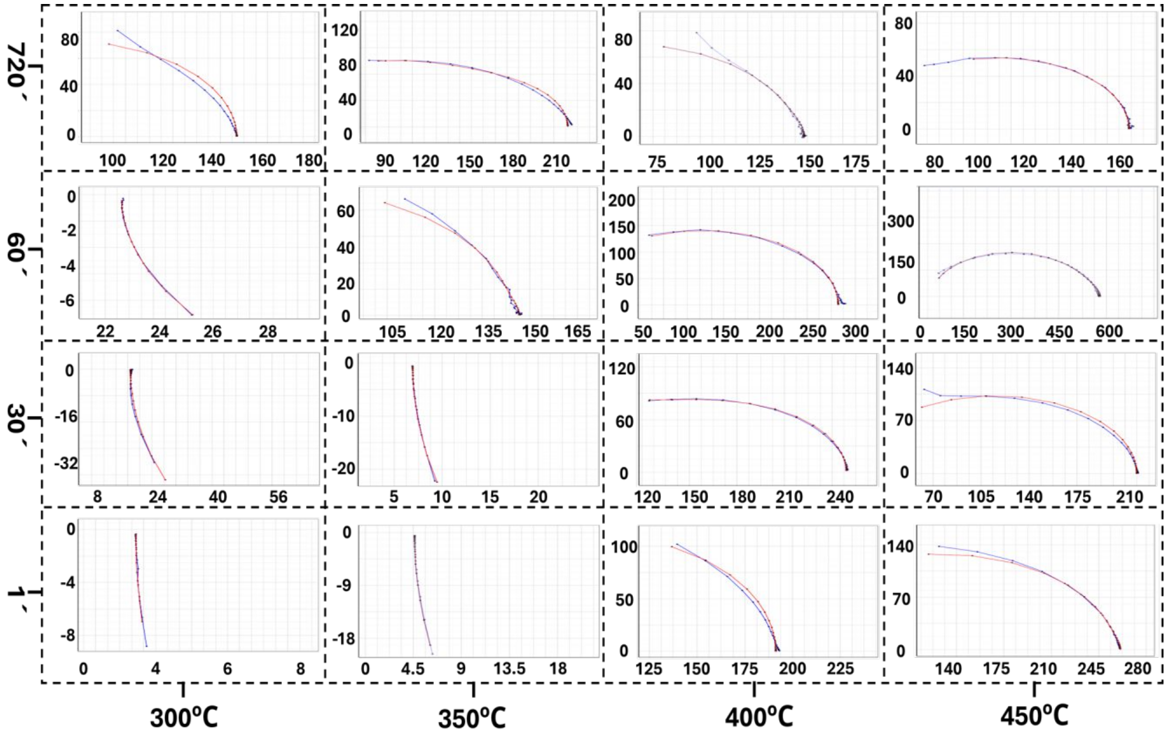

Figure 3. The frequency-dependent response of each sample (blue), along with their corresponding Z-fitted curves (red) for the frequency range (0Hz to 3-5MHz).

Figure S4

The following circuit was suggested for our disconnected Pt nano islands in which the inherent capacitive nature of the Pt nano islands is represented by constant phase element (CPE), which gives more realistic values to the system and has been shwon parallel to the inherent resistance of the Pt nanonetworks. While the contact resistance has been shown in the series.

The suggested circuit for our interconnected Pt nanonetworks was the same as we used at 3MHz frequency, shown in the Figure S11.

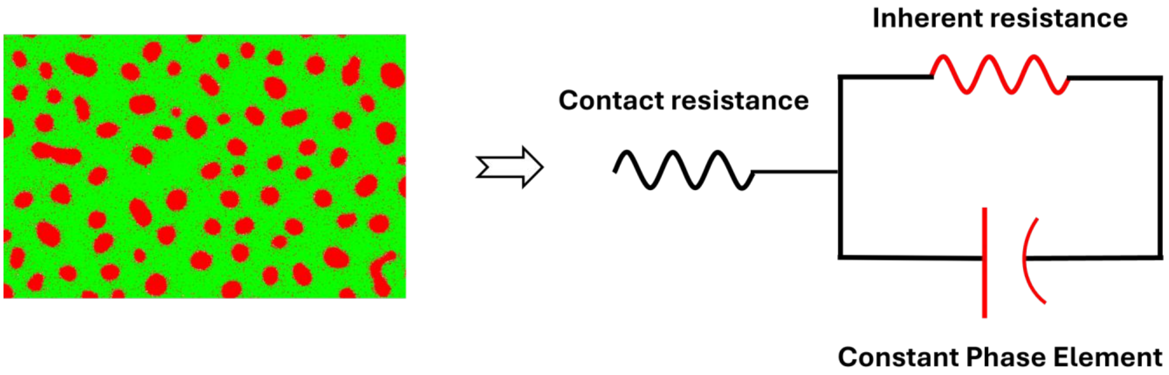

Figure 4. Suggested equivalent circuit for the electronically interconnected Pt nanonetworks for the frequency range (0Hz to 30MHz)

Figure S5

The blue graph displays the original data collected from the samples at frequencies ranging from 0 Hz to 30 MHz. In contrast, the red graph represents the Z-fitting based on the equivalent circuit model as shown in Figure S11 and Figure S14. Each Nyquist plot shows the impedance data, with the real and imaginary components plotted on the x-axis and y-axis, respectively, both in ohms (Ω).

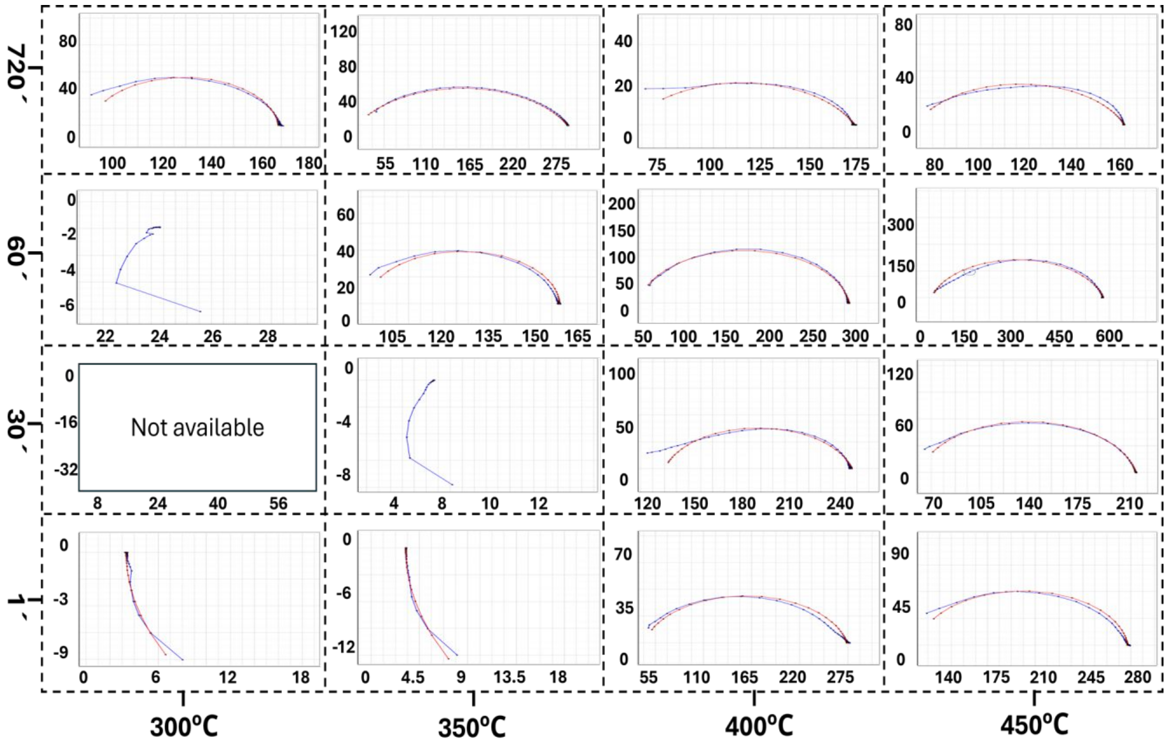

Figure 5. The frequency-dependent response of each sample (blue), along with their corresponding Z-fitted curves (red) for the frequency range (0Hz to 30MHz).

Figure S6

Real-time imaging of flexible nanonetworks on a polyimide substrate under varying bending diameters. 0 mm shows zero bending condition.

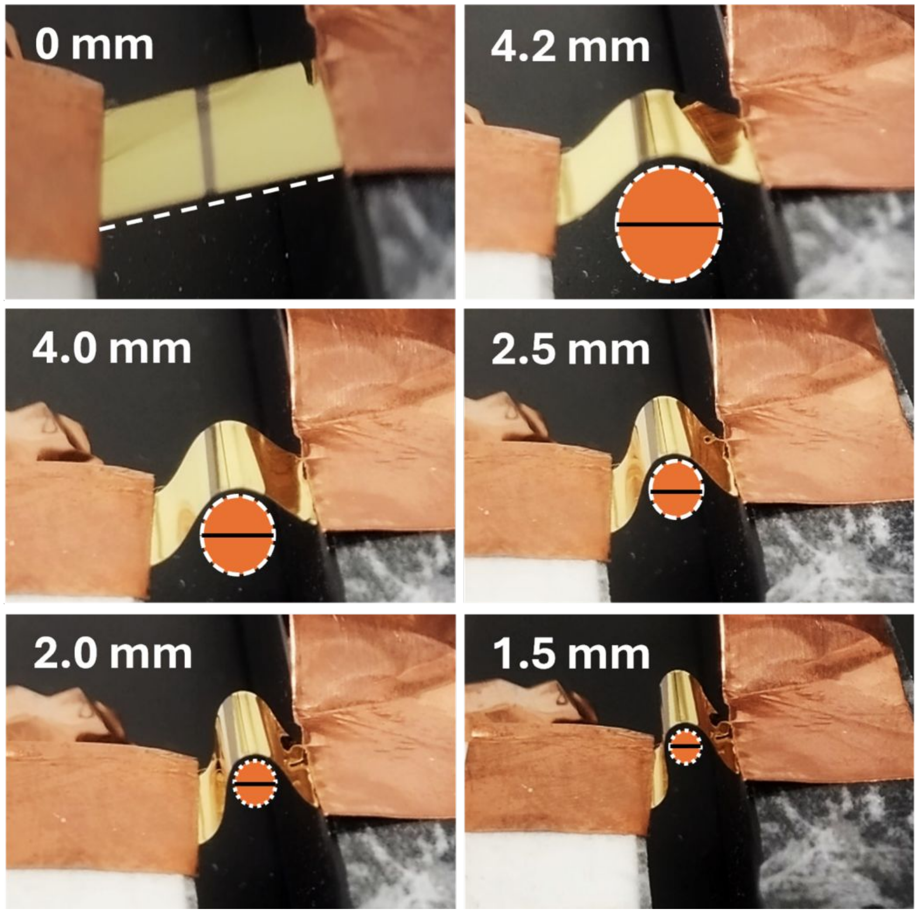

Figure 6. Real time images of flexible nanonetworks over polyimide substrate at different bending diameters.

Figure S7

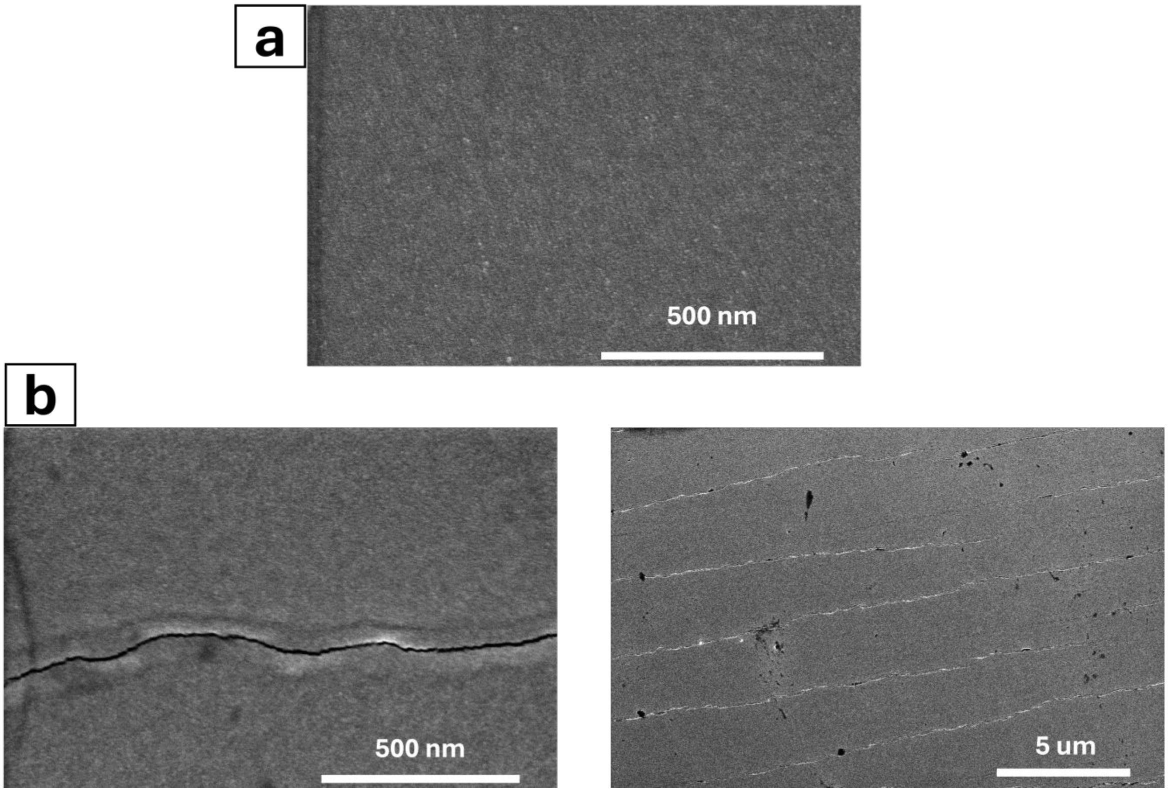

Figure 7. FE-SEM images of PtCe alloy film over PI, (a) 0 bending cycle at scale 500 nm, after 20 bending cycles (b), at scales of 500nm and 5 um.

Figure S8

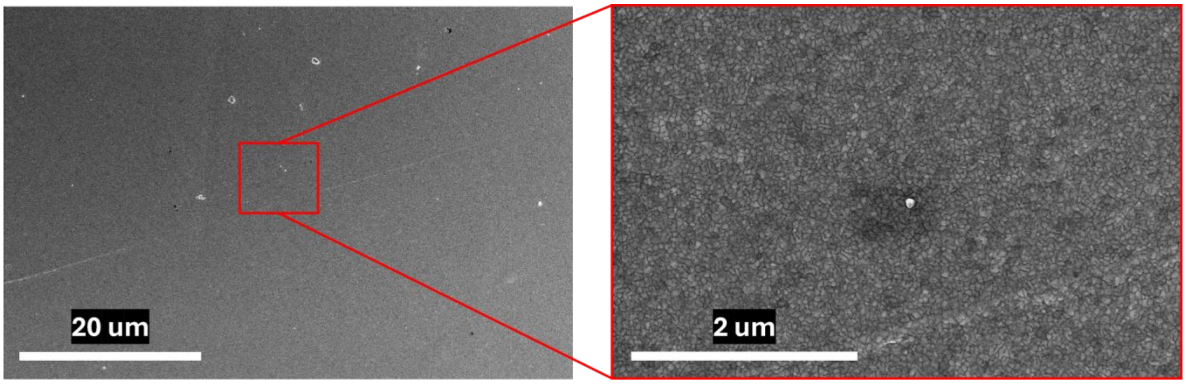

Figure 8. FE-SEM images of Au/Ti terminals at 0 bending cycles.

Figure S9

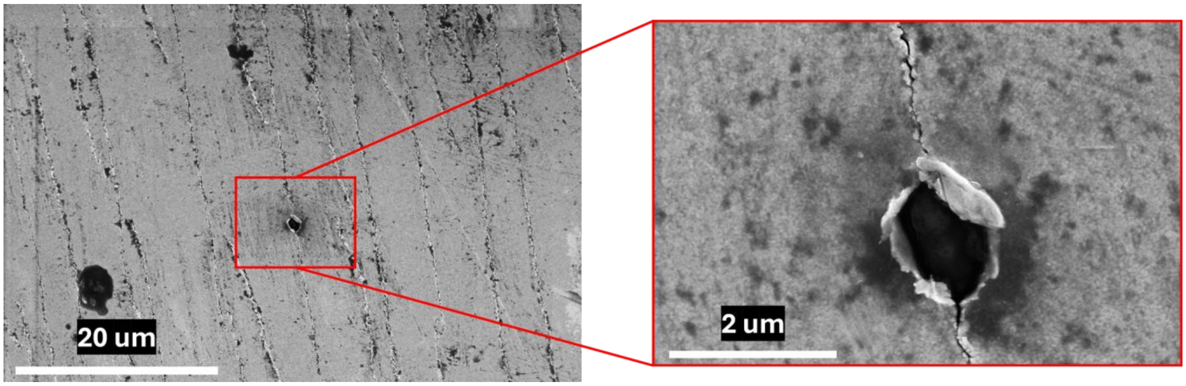

Figure 9. FE-SEM images of Au/Ti terminals after 1000 bending cycles.

Figure S10

The SEM and EDX images of the as-deposited Pt-Ce alloy 50nm thin film over the Si substrate illustrate the uniform distribution of Pt and Ce in the alloy form all over the surface of the Si substrate.

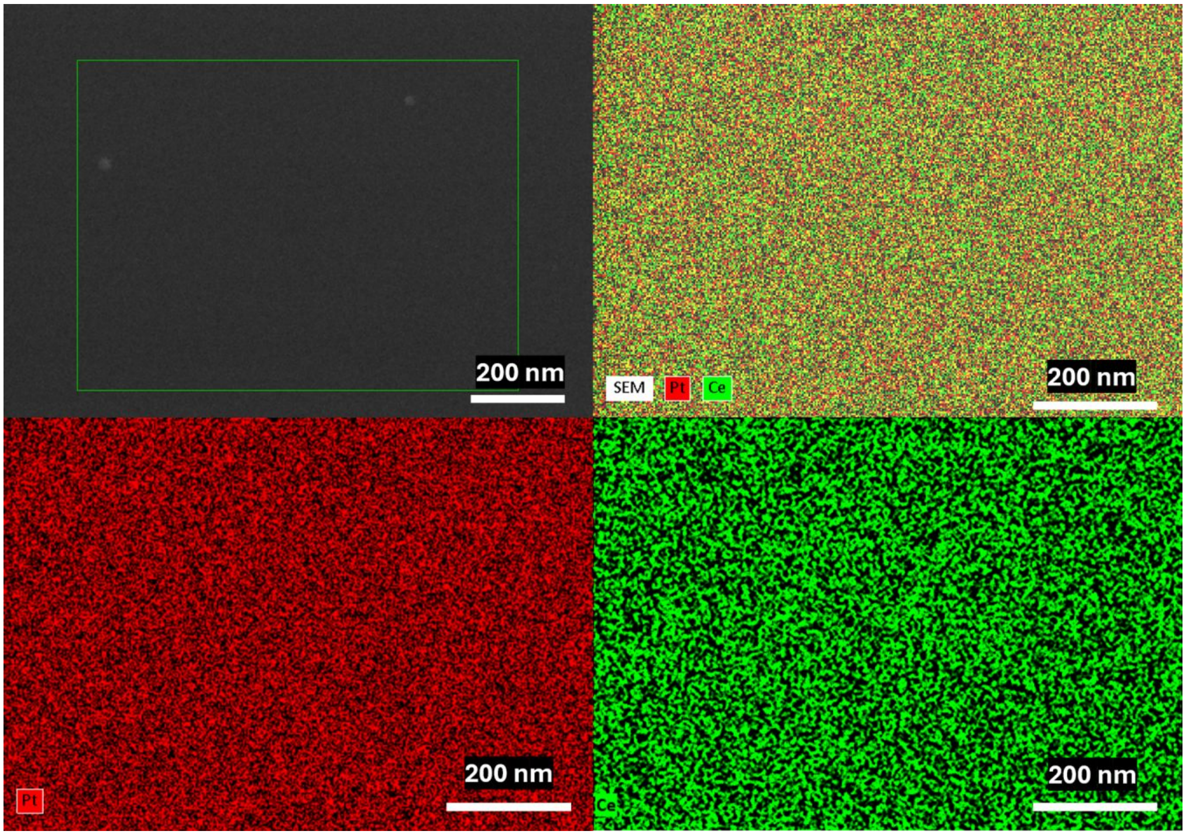

Figure 10. FE-SEM and EDX images of as-deposited 50nm thin film of Pt-Ce alloy over Si substrate.

Figure S11

The mechanism behind the electron beam evaporation of the Pt-Ce alloy ingot to deposit a thin film of 50nm over the Si substrate at a back pressure of 10^{-5} Pa via eelectron-beam evaporator (MB-501010). The directed electron beam falls over the target to sublimate it for attaining a very thin layer of it. The thickness of the film is monitored by quartz crystal to accomplish accurate thickness.

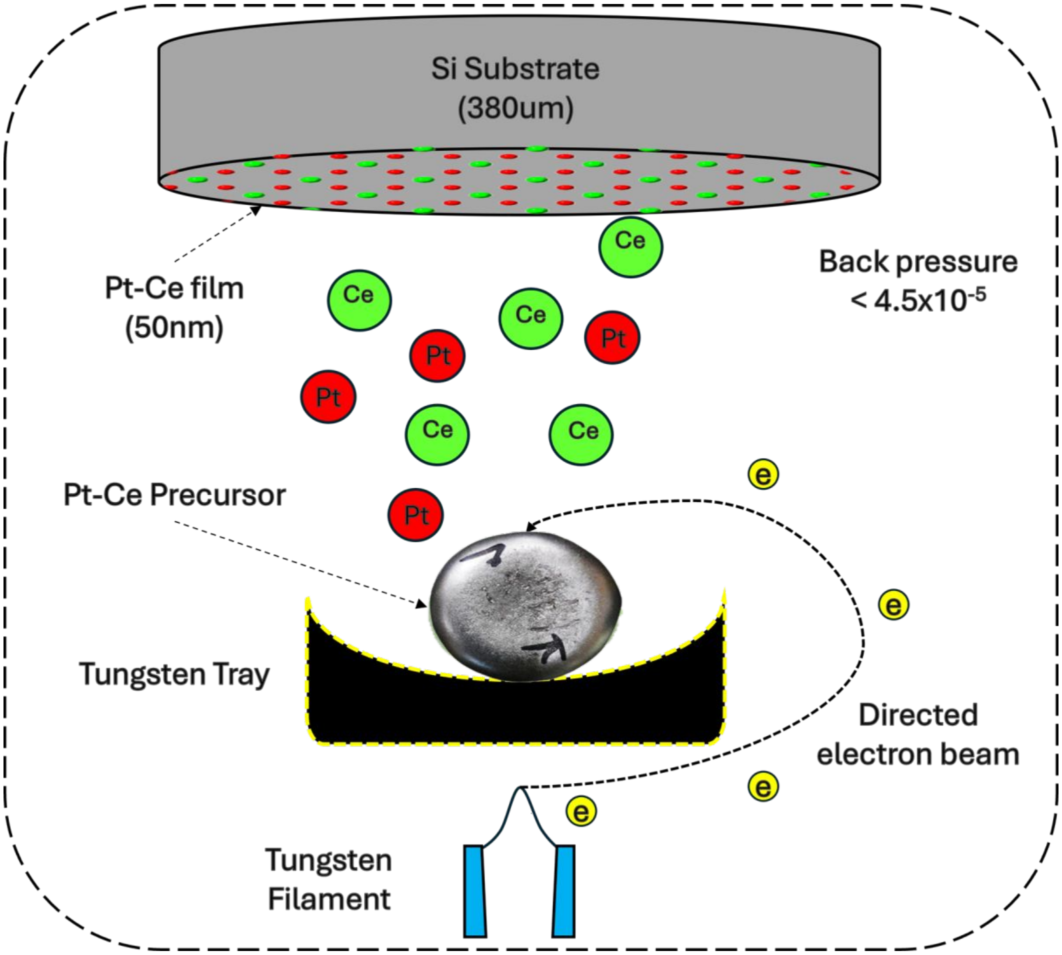

Figure 11. Electron beam evaporation mechanism

Figure S12

The grazing index XRD was also conducted on the as-deposited 2D thin films. GI-XRD is favorable for thin films since most of the reflection attained is from the thin film itself rather than the substrate. This is because the penetration depth of X-rays in the GI-XRD is around 200nm. The observed reflections in the GI-XRD analysis were identified as originating from the Pt-Ce alloy.

Grazing incidence XRD was performed on the atmosphere-treated 2D thin film as well, revealing distinct reflections from Pt metal and CeO_2 instead of the Pt-Ce alloy. These findings confirm the transformation of the Pt-Ce alloy thin film into the atmosphere-treated Pt#CeO_2 thin film, characterized by Pt nanonetworks.

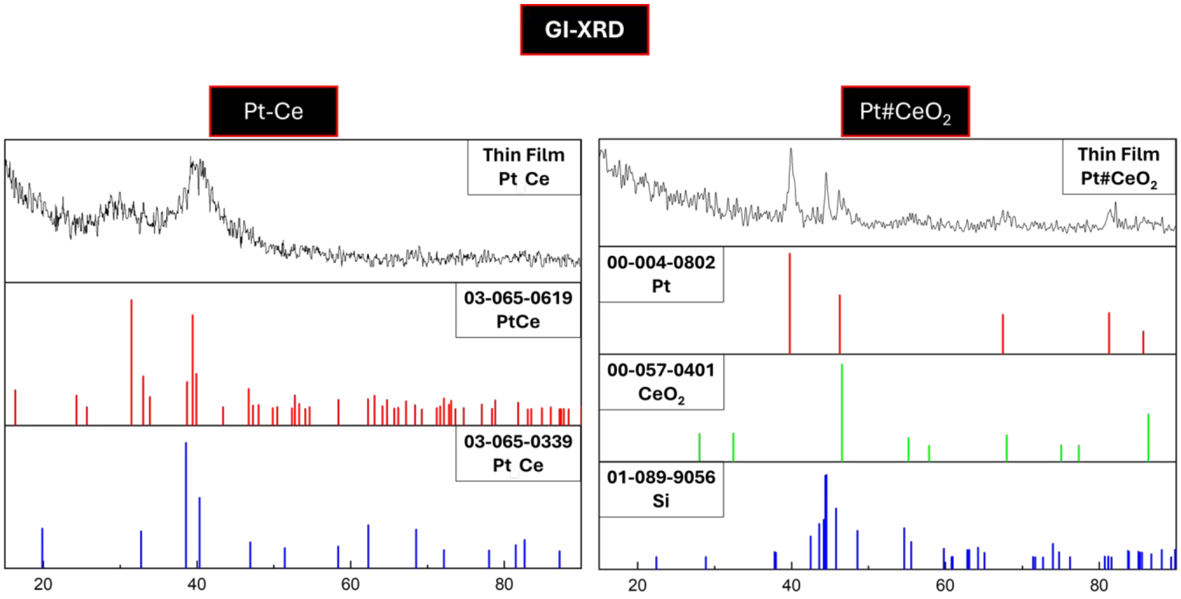

Figure 12. GIXRD of the as-deposited Pt-Ce film (left) and atmosphere-treated Pt-Ce film (right).

Figure S13

SEM analysis revealed nanophase separation in the atmosphere-treated Pt–Ce film, showing fibrous Pt phases forming a 2D nanonetwork with an average width of 33 nm. This network exposed the Si substrate between Pt phases, creating a negative image of Pt distribution. Ce and O were primarily concentrated over the surface. The resulting self-organized nano-pattern on the Si surface is referred to as Pt#CeO_2/Si. These findings exaclty resembles with our previously published work, reference number 13 of the main article.

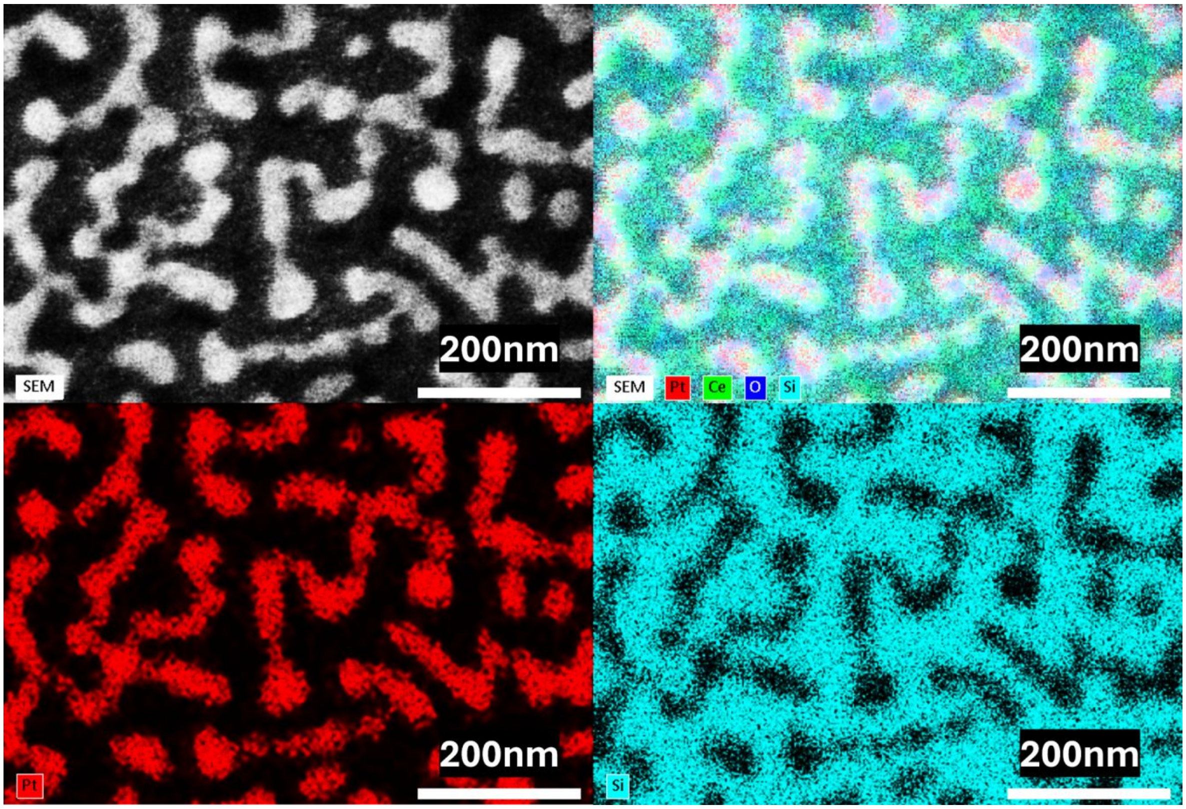

Figure 13. FE-SEM and EDX images of atmosphere-treated 50nm thin film of Pt-Ce alloy over the Si substrate.

Figure S14

Atomic force microscopy (AFM) was also utilized to examine the atmosphere-treated films and track the nanonetworks' paths through electrostatic forces. The Gwyddion software was employed to evaluate the height profile, which measured approximately 4 nm to 5 nm. The Pt nanonetworks are represented by the bright nanomeshes in the AFM images. For the KPFM images please see the reference number 13 of the main article.

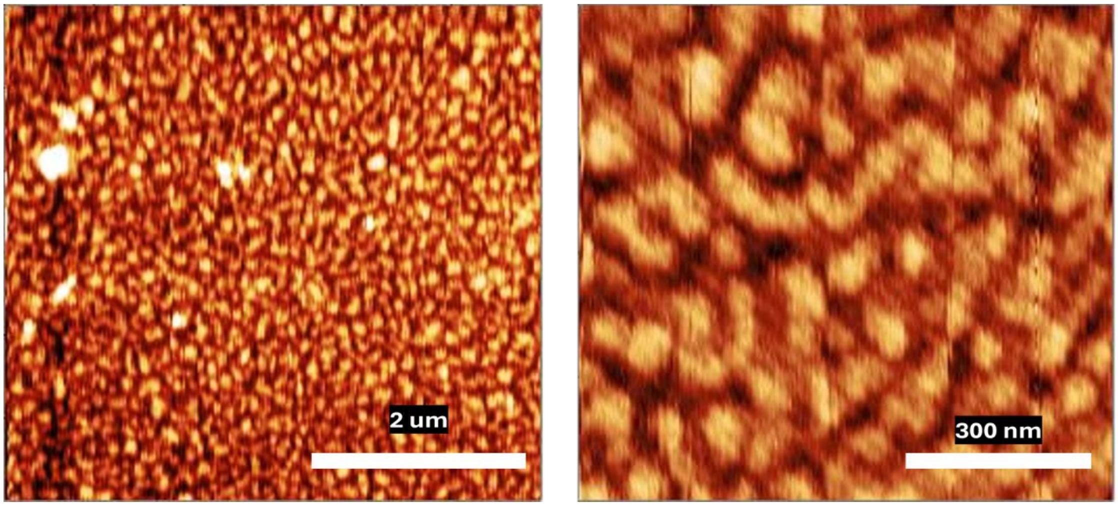

Figure 14. AFM images of the atmosphere-treated 50nm thin films of Pt-Ce alloy over the Si substrate.

Figure S15

A pair of gold-titanium (Au-Ti) terminals were deposited onto the post-treated film surface over Si substrate (Pt-CeO_2/Si), with a 25 µm terminal gap maintained using a 25 µm thick aluminum wire. The deposition was carried out using an electron beam evaporator (UEP-3000BS).

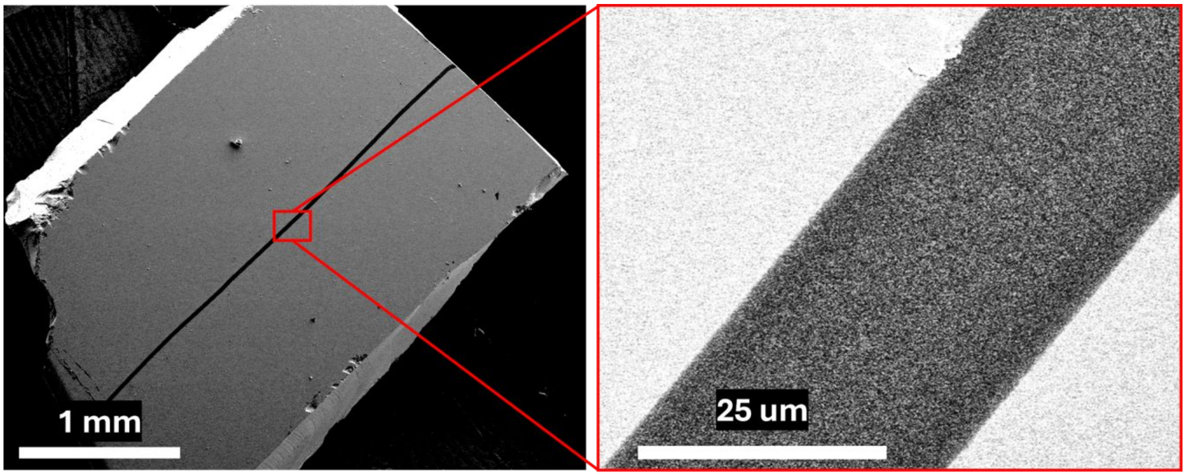

Figure 15. FE-Sem images of the gold-titanium (Au-Ti) terminals with a separation of 25 microns.

Figure S16

The 2-probe method was utilized for the impedance analysis of the atmosphere-treated 50nm Pt-Ce thin films over the Si substrate as shown in the figure below.

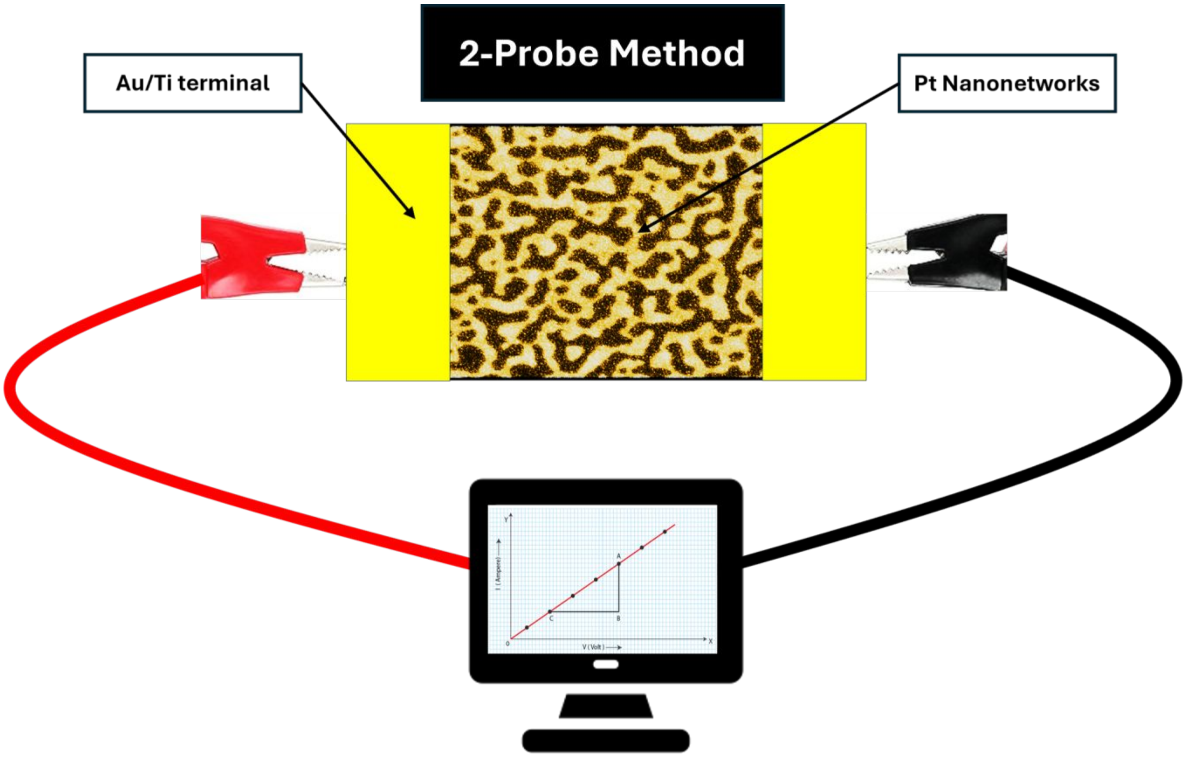

Figure 16. 2-Probe method setup for the impedance analysis

Figure S17

FE-SEM images confirmed the uniformity of the as-deposited Pt-Ce film on the polyimide (PI) substrate, at a very high magnification.

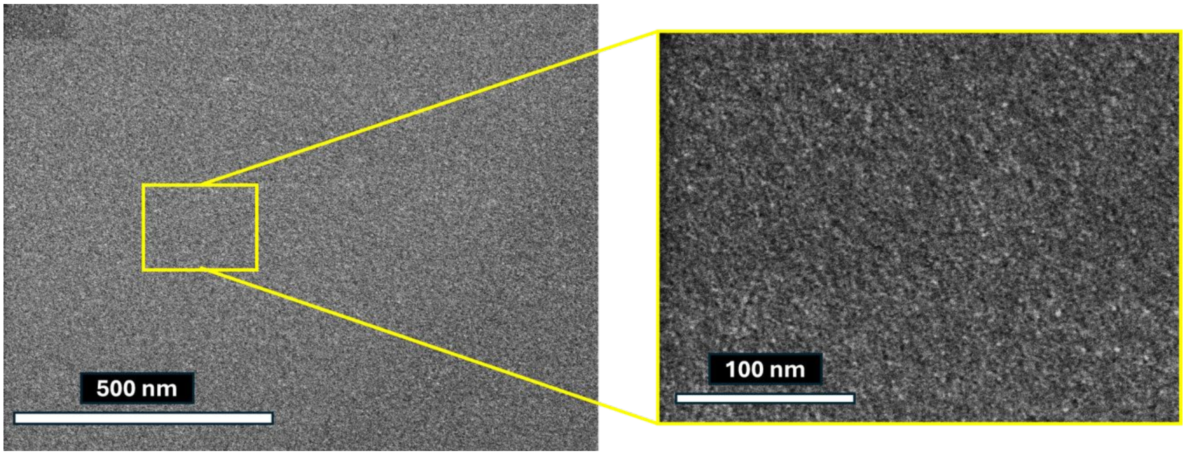

Figure 17. FE-SEM of as-deposited 50nm thin film of Pt-Ce alloy over the flexible PI substrate.

Figure S18

FE-SEM images at a very high magnification confirmed the uniformity of the atmosphere-treated Pt-Ce film on the polyimide substrate. The bright contrast area revealed the formation of the Pt nanonetworks over the flexible PI substrate.

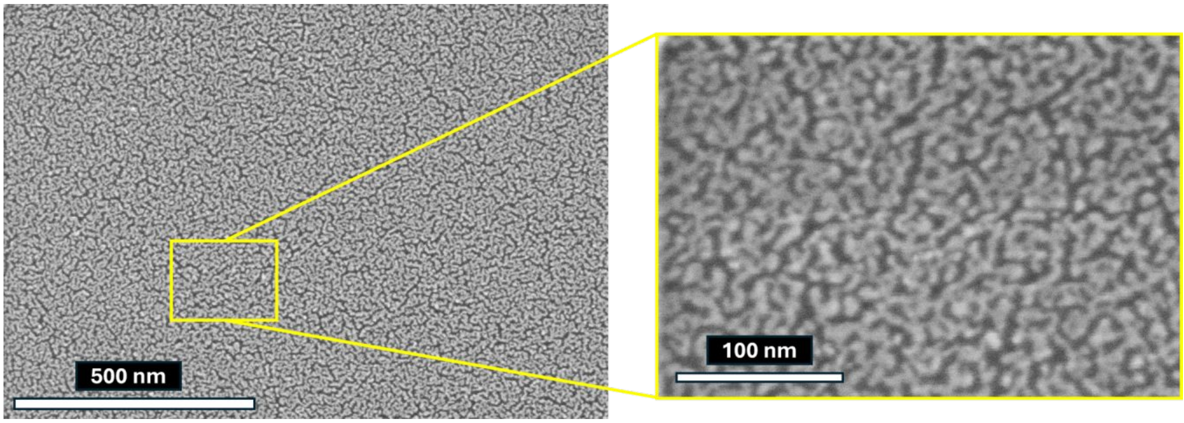

Figure 18. FE-SEM of atmosphere-treated 50nm thin film of Pt-Ce alloy over the flexible PI substrate.

Figure S19

EDX images at high magnification confirmed the formation of Pt nanonetworks on the flexible PI substrate, represented as green nanonetworks in the image.

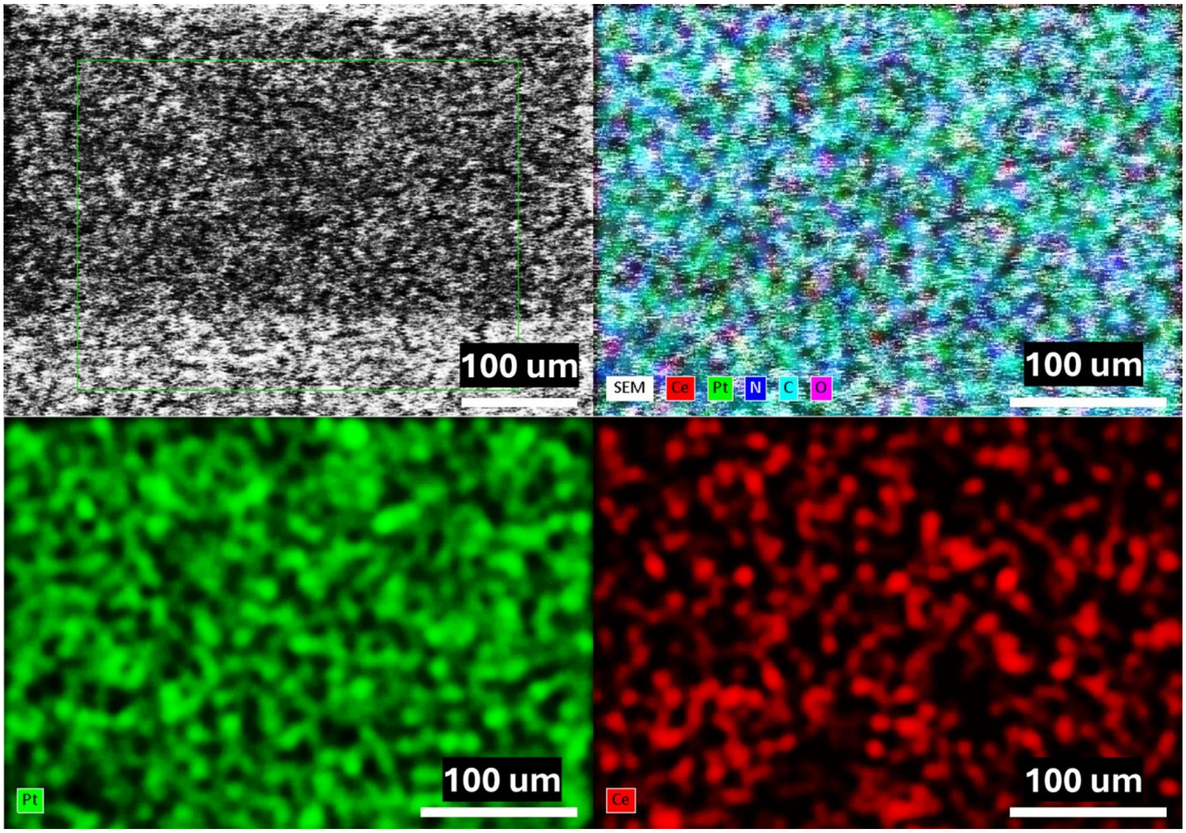

Figure 19. EDX images of the atmosphere-treated 50nm thin film of Pt-Ce alloy over the flexible PI substrate.

Figure S20

The 4-probe method was utilized for the impedance analysis of the atmosphere-treated 50nm Pt-Ce thin films over the Polyimide (PI) substrate as shown in the figure below.

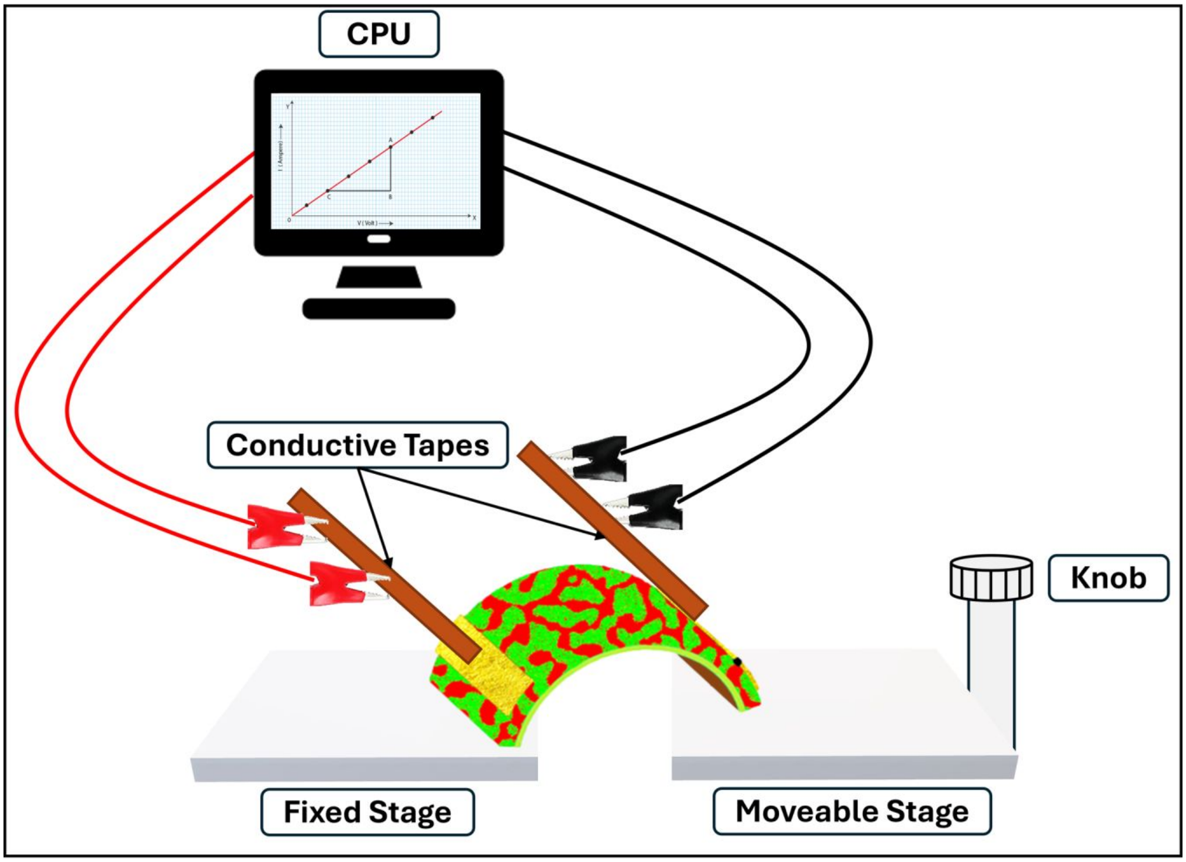

Figure 20. 4-Probe method setup for the impedance analysis
